# Integration of Acoustic Radiation Force and Optical Imaging for Blood Plasma Clot Stiffness Measurement

**DOI:** 10.1371/journal.pone.0128799

**Published:** 2015-06-04

**Authors:** Caroline W. Wang, Matthew J. Perez, Brian P. Helmke, Francesco Viola, Michael B. Lawrence

**Affiliations:** 1 Department of Biomedical Engineering, School of Engineering and Applied Science and School of Medicine, University of Virginia Health System, Charlottesville, Virginia, United States of America; 2 HemoSonics, LLC, Charlottesville, Virginia, United States of America; University of Michigan, UNITED STATES

## Abstract

Despite the life-preserving function blood clotting serves in the body, inadequate or excessive blood clot stiffness has been associated with life-threatening diseases such as stroke, hemorrhage, and heart attack. The relationship between blood clot stiffness and vascular diseases underscores the importance of quantifying the magnitude and kinetics of blood’s transformation from a fluid to a viscoelastic solid. To measure blood plasma clot stiffness, we have developed a method that uses ultrasound acoustic radiation force (ARF) to induce micron-scaled displacements (1-500 μm) on microbeads suspended in blood plasma. The displacements were detected by optical microscopy and took place within a micro-liter sized clot region formed within a larger volume (2 mL sample) to minimize container surface effects. Modulation of the ultrasound generated acoustic radiation force allowed stiffness measurements to be made in blood plasma from before its gel point to the stage where it was a fully developed viscoelastic solid. A 0.5 wt % agarose hydrogel was 9.8-fold stiffer than the plasma (platelet-rich) clot at 1 h post-kaolin stimulus. The acoustic radiation force microbead method was sensitive to the presence of platelets and strength of coagulation stimulus. Platelet depletion reduced clot stiffness 6.9 fold relative to platelet rich plasma. The sensitivity of acoustic radiation force based stiffness assessment may allow for studying platelet regulation of both incipient and mature clot mechanical properties.

## Introduction

The formation of a blood clot through the interaction of platelets, fibrin polymers, and the vessel wall is the primary mechanism by which the body arrests blood loss after injury. Platelet activation, fibrinogen polymerization and fibrin fiber crosslinking alter the mechanical properties of blood within minutes from those of a viscous fluid to that of a viscoelastic gel [1,2]. Effective blood clot formation therefore requires an increase in material stiffness, a characteristic that can be correlated with clinical coagulopathies [3,4].

*Ex vivo* blood clot mechanical properties have been correlated with a number of disease states. Inadequate clot stiffness, manifested as a 'soft' clot, is characteristic of hemorrhagic disorders such as hemophilia and has been correlated with surgical bleeding in cardiopulmonary bypass [5,6]. 'Hard' clots, by contrast, have been linked to the formation of thrombi in stroke, myocardial infarction, and deep vein thrombosis [1,7–10].

The relationship between clot mechanics and vascular diseases underscores the importance of identifying the contributory roles of platelets and fibrin to hemostasis. Several *ex vivo* mechanical tests of clot stiffness have successfully transitioned into the clinic, most notably the thromboelastogram (TEG) and rotational thromboelastometry (ROTEM). Measurement of bulk clot stiffness by either the TEG or ROTEM can be structured as a differential test to assess the relative contributions of platelets and plasma factors to overall clot stiffness [11–13].

There are a number of new technologies that are being developed based on diverse approaches to look at the dynamics of blood clotting both *in vivo* and *ex vivo*. Dynamic light scattering analysis has been used to measure *in vivo* the clotting of blood by comparing motion of RBCs in flow or in stasis [14] and laser speckle rheology has been used to correlate changes in light scattering speckle intensity with viscoelastic property changes *ex vivo* [15]. A novel approach that combines laser detection of material motion and mechanical oscillation of the blood sample is RheoSpectris [16]. Ultrasound imaging has been used for detection of material strains in tissue following external application of force [17,18]. In addition there are multiple microelectromechanical systems (MEMS) technologies that incorporate microcantilever or piezoelectric approaches to measure the changes in material properties of blood *ex vivo* [19,20]. Recently developed high resolution measurements of individual platelet contractility and fibrin fiber stiffness have given additional insight into the cellular and molecular basis of clot mechanics, though single cell and molecule assessments have yet to be linked to global or bulk rheological properties of clots [9,21]. The majority of viscometric assessments of clot rheology require removal of a blood sample from the subject for analysis and therefore may not capture fully the pathophysiology of an arterial or venous clot in situ. Within this admittedly severe limitation, *ex vivo* approaches to studying blood clotting can give significant insight into detailed molecular and cellular mechanisms that regulate coagulation.

In contrast to approaches to assess *ex vivo* blood clot stiffness based on either light scattering or mechanical testing, ultrasound can assess tissue stiffness by generation of acoustic radiation force (ARF), which transfers momentum from the ultrasound waves to the propagating medium and any embedded reflective or absorptive objects [22,23]. ARF has been previously used to characterize the viscoelastic properties of various biological tissues including the liver, tumor tissue, and the vitreous body of the eye [24–27]. In a more recent development to assess viscoelastic properties of blood, ultrasound ARF is coupled with echo time delay estimation of red blood cell displacement in a technique called sonorheometry [28–30]. Other technologies that employ ultrasound in their approach to detect material properties of blood include supersonic shear imaging [31].

**Fig 1:**
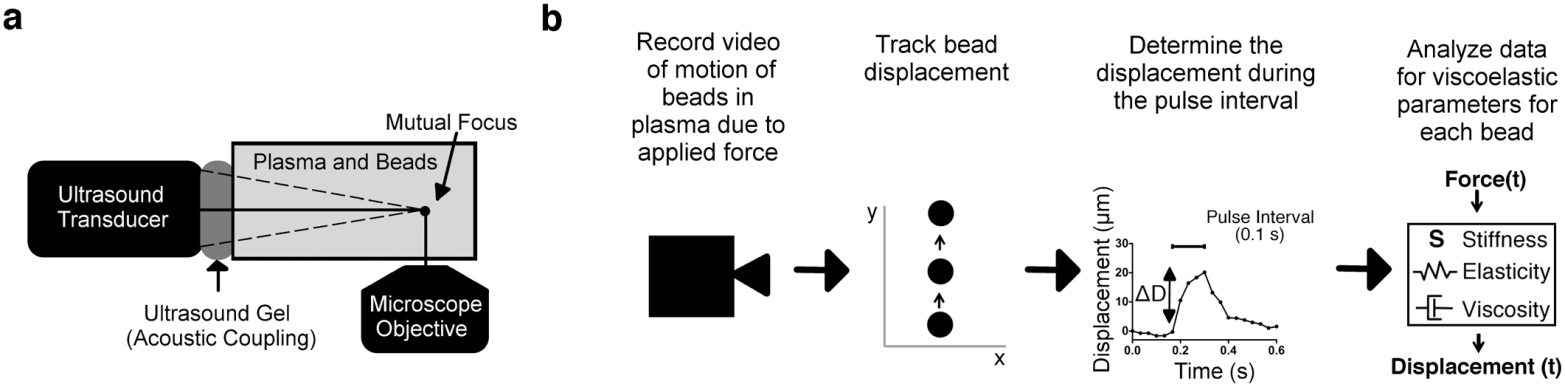
Acoustic Radiation Force (ARF) based clot stiffness measurement. (**a**) The ARF based measurement setup used a 10 MHz ultrasound transducer to apply ARF to blood plasma in which beads were suspended. A microscope with 10 X objective and video camera had a mutual focus with the ultrasound transducer so application of ARF to the sample was imaged. (**b**) To assess blood plasma stiffness: 1) Video microscopy of the blood plasma sample was collected during application of ARF, 2) The bead motion due to applied ARF was tracked by an ImageJ program, 3) Displacements during the ARF pulse interval were determined, and 4) The displacement data was analyzed for the sample’s viscoelastic parameters.

Here we describe the development of an optical ARF-based method of strain application to blood plasma clots such that the contributions of platelets and fibrin to its mechanical properties can be individually assessed (Fig 1a). Acoustically reflective microbeads were embedded in the clot to act as force transducers and strain gauges. Ultrasound ARF was then used to induce bead displacement within a plasma sample that was then tracked by video microscopy to evaluate clot stiffness (Fig 1b). The focal point of the ultrasound transducer corresponded to a microliter-sized region of a blood clot that was positioned away from container walls so that it acted on a relatively small numbers of platelet clusters within the 3D clot matrix. ARF was controlled to generate rapid and highly transient (subsecond) mechanical strains of a few 10s of microns to quantify local plasma clot stiffness. Because the method perturbed a small clot region and applied small strains, it measured blood clot stiffness at an intermediate length scale between the milliliter volumes of conventional rheometric approaches and single molecule and cell approaches such as optical tweezers or atomic force microscopy (AFM). ARF-based assessment of clot stiffness may therefore be useful for investigating the cooperative role of platelet signaling and fibrin dynamics in the elastic transformation of a blood clot.

## Methods

### Ethics statement

All participants provided written signed informed consent after explanation of study procedures. Experiments and study protocol as described in IRB-HSR # 12600 were approved by the Institutional Review Board for Health Sciences Research of the University of Virginia.

### Experimental setup

The experimental setup (Fig 1a) consisted of a platform, which positioned the ultrasound transducer relative to the microscope objective at the transducer focal distance. The sample was held in place over the objective and acoustically coupled to the transducer with ultrasound gel (Aquasonic, Parker Laboratories, Fairfield, NJ, USA). The sample container was slightly larger than the transducer diameter in order to minimize beam interference with the container walls. A 2.54 cm square polystyrene box (8.2 mL) (Ted Pella, Redding CA, USA) was used to hold the fluid or plasma sample. An acoustically absorbent V-shaped polydimethylsiloxane form (see S1 Fig) was inserted into the box to minimize acoustic reflections and reduce the required sample volume to 2 mL. A 10 MHz single-element focused transducer (model IBMF103, NDT Systems, Huntington Beach, CA, USA) was coupled to the sample holder with ultrasound gel. The 10 MHz transducer had a diameter of 9.5 mm and a focal length of 19 mm. The transducer and sample holder were held in place by a custom stage mounted on an air table. The sample holder was mounted above a microscope (Diaphot 300, Nikon, Melville, NY, USA) with a 10 X microscope air objective, which permitted focusing into the sample at varying depths. For optimal foci alignment of the ultrasound beam with the objective plane of focus, the optical focal plane was fixed at 1300 μm above the sample holder surface for all stiffness measurements. The effect of different optical focal depths on bead displacement was investigated and the analysis is included in S2 Fig. The motion of beads dispersed within the sample was acquired at 30 fps by a video camera (Canon Vixia HF S21, Canon, Melville, NY, USA) mounted to the microscope camera port. As beads experiencing ARF move more slowly than material shear waves due to their size relative to fundamental material components (e. g. molecules, fibers), standard frame rate (30 fps) video microscopy can be used to assess bead trajectories with a high degree of precision.

### An acoustic beamforming model

The ultrasound beam intensity and pressure profiles were modeled using Field II (http://field-ii.dk/), a well characterized ultrasound simulation tool [32]. The example code provided by the designer for producing intensity and pressure profiles, calc_int.m, was modified for the specific transducer specifications and settings used in this study: transducer center frequency (10 Mz), transducer radius (9.525 mm), focal length (19.05 mm), cycles per pulse (8), pulse repetition frequency (7.5 kHz), number of elements (1). The transducer aperture was modeled using the function xdc_concave. The intensity was set to 202 W/m^2^ in order to match the peak pressure 2.5MPa to that which was measured with the hydrophone. The program then generated simulated beam intensity and pressure profiles.

### Bead tracking and processing of displacement data

Two methods of single particle tracking implemented in the image processing software ImageJ (NIH, Bethesda, MD, USA) were used to quantify bead displacement due to incident ARF: a manual particle tracking protocol and an automated tracking algorithm [33]. Manual tracking was used when bead velocities were greater than 400 μm/s and the automated algorithm was used in all other cases. At high velocities manual tracking was more effective due to edge detection limitations in the automated tracking algorithm. The automated tracking algorithm utilized a cross-correlation technique to measure sub-pixel and sub-micron displacements [34].

Manual tracking was performed using the point tool in ImageJ with the “auto-measure” and “auto-next slice” features turned on. Determination of the elastic and viscous parameters of the Kelvin-Voigt viscoelastic model from position data was performed using curve fitting with the built-in function lsqcurvefit in the computational software Matlab (Natick, MA, USA) where the lower and upper bounds for viscosity (μ) and elasticity (k) were set to 0.1 and 10 *pN·s·μm* ^*-1*^ and 0.1 and 10,000 *pN·μm* ^*-1*^, respectively.

Because beads like those used in optical ARF move much more slowly than material shear waves due to bead size relative to fundamental material components (e. g. molecules, fibers), standard frame rate (30 fps) video microscopy can be used to assess ARF induced bead motion.

### Force calibration experiments

For determining stiffness and for modeling viscoelasticity, the magnitude of force applied must be known. The ARF was estimated by tracking beads and analyzing their trajectories and terminal velocities in known viscosity fluids. Stokes’ law was applied to determine the force on an object moving through a fluid of known viscosity. We used 3 fluids with known viscosities, two NIST traceable viscosity standards (S3 and S6) (Cole Parmer, Court Vernon Hills, IL, USA) and blood plasma to calibrate ARF on the bead.

To estimate force, 15 μm diameter latex polystyrene beads were suspended in the fluid samples Polybead, Polysciences, Warrington, PA, USA). The S3 and S6 fluids have precisely known viscosities of 4.063 cP and 8.743 cP, respectively, at 25°C. At room temperature blood plasma has a viscosity of 1.6 ± 0.1 cP [35].

The sample holder was filled with a viscosity standard in which beads were suspended. The 10 MHz transducer used to generate ARF was triggered in all experiments (except where noted) by a custom amplifier circuit connected to a Macbook 2,1 laptop computer (Apple, Cupertino, CA) running a Matlab/C++ program that controlled the ultrasound transducer firing pattern. A 0.8 μs pulse length (8 wave cycles) was applied. We define the rate of pulse generation used to create an impulse sequence by a pulse repetition frequency (PRF). Pulses were generated at a PRF of 7.5 kHz over a pulse train interval of 0.5 s. Pulse train intervals were separated by 6 s of rest. In place of the custom amplifier circuit, a waveform generator and power amplifier were substituted to increase dynamic range in selected experiments.

To investigate the effect of changing the PRF on bead displacement the PRF was varied in the viscosity standards. PRFs of 15, 7.5, 3.75, and 1.875 kHz were used at a time interval of 0.5 s. The result was a total of 7500, 3750, 1875, and 937 pulses fired per burst for each respective condition.

### Measurement and analysis of ultrasound attenuation in viscosity standards and in agarose gels

A 10 MHz ultrasound transducer for transmitting ultrasound signals and a signal receiver, either a bullet hydrophone (Onda Corporation, Sunnyvale, CA) or 10 MHz ultrasound transducer, were immersed in a degassed water bath. A two-axis micron precision stepper motor controlled the relative positions of the transmit transducer and the signal receiver (hydrophone or receive transducer). For measurement of ultrasound attenuation in the viscosity standards and in agarose, sample holders with varying acoustic path lengths were built (see S3 Fig). An arbitrary waveform generator AFG 3022B (Tektronix, Beaverton, OR, USA) and 55dB amplifier ENI A-150 (E & I, Rochester, NY, USA) was used to drive the transducer. A Lecroy 334A oscilloscope (Lecroy, Chestnut Ridge, NY, USA) collected waveforms from each transducer or hydrophone that were transferred by GPIB to computer for analysis. The attenuation was determined by comparing ultrasound signal amplitudes at different path lengths of viscosity standard and using analysis techniques described in a later section.

The total path length between the transmit and receive transducer or hydrophone was determined by the measurement of the time delay between the pulse transmission and the pulse receipt with the speed of sound in water (1497 m/s) [36]. Change in signal voltage due to signal path length within the material was determined by the average absolute value of the waveform. A trend of reduced signal voltage with material thickness was measured. The voltage attenuation data was used to form a system of equations specific for each thickness of material and solved for the attenuation coefficient of the material using the Matlab function lsqnonlin. The attenuation of water was 0.0022 *dB·cm* ^*-1*^·*MHz* ^*-1*^ [37].

### Agarose gel formation

Agarose gel was made with UltraPure Agarose (Life Technologies, Grand Island, NY, USA). The agarose was added to Tris-Acetate-EDTA (TAE) buffer in proportions to generate a 0.5 wt % gel in an Erlenmeyer flask. The solution was heated for 30 s in the microwave and then poured into either a 2.54 cm or 10 cm square polystyrene box to a volume appropriate for the thickness of gel desired (8.2 mL or 500 mL, respectively). Agarose was allowed to cool for 1 h before removal from the mold and measurement of attenuation.

### Agarose gel and blood plasma bead displacement measurement

The agarose gel was made as described above with the exception that 10 μL of 5 μm carboxylated YG fluorescent beads (2x10^6^)(Fluoresbrite, Polysciences, Warrington, PA, USA) were added. Similarly PRP was doped with 10 μL of 5 μm carboxylated YG fluorescent beads before initiating clotting. Both agarose and plasma gelled in molds for 1 h to maximize stability and permit transfer to a gel holder mounted in a water bath on the stage of the microscope. In this way acoustic attenuation was minimized to increase force transmittal and permit analysis of the much stiffer agarose gel. A ziplock bag was used to enclose each sample in its appropriate buffer (TAE in the case of agarose or Dulbecco's Phosphate Buffered Saline (DPBS) in the case of plasma). The sample was then aligned at the mutual focus of the 10 MHz transducer and a 40 X water immersion microscope objective. The fluorescent beads allowed imaging in a turbid gel. For generation of ARF the transducer was driven by an arbitrary waveform generator AFG 3022B (Tektronix, Beaverton, OR, USA) and 55dB amplifier ENI A-150 (E & I, Rochester, NY, USA).

### Bead sedimentation velocity measurement

Bead sedimentation velocity was determined experimentally in citrated platelet rich plasma (PRP) by measuring bead time of flight over the thickness of a gasket in a flow chamber. The gasket thickness allowed for precise measurement of distance travelled due to gravity from one surface to the other.

### Blood clotting experiments

Blood was collected from two healthy volunteers using the protocol described in IRB-HSR # 12600 in 2.7 mL citrated 3.2% Vacutainer tubes (BD, Franklin Lakes, NJ, USA). Blood drawn by venipuncture resulted in more variable levels of platelet activation, hence the use of Vacutainers for sample acquisition. The blood was centrifuged at 100 x G for 20 min to separate phases of PRP, buffy coat, and red blood cells. To obtain PRP, the supernatant of the centrifuged samples was collected by pipette. An additional centrifugation step of 2000 x G for 40 min was used to obtain platelet poor plasma (PPP). Samples consisted of 2 mL of fluid volume mixed with 0.5 x 10^6^ / mL of 15 μm diameter latex polystyrene beads (Polybead, Polysciences, Warrington, PA, USA). Plasma samples were pipetted into the experimental sample chamber. To initiate clotting, samples were recalcified with CaCl_2_ (Sigma, St. Louis, MO, USA) to bring the PRP to a concentration of 9.525 mM CaCl_2_. Kaolin (JT Baker, VWR, Radnor, PA, USA) was added to a final concentration of 5 μg/mL, except where otherwise noted. Clotting and the ARF pulsing sequence were initiated within 15 s of kaolin addition. Particle motion within the sample was recorded for 30 s intervals every 3 minutes throughout the initial 30 min of clotting for each sample.

## Results

### Spatial distribution of acoustic radiation force

**Fig 2:**
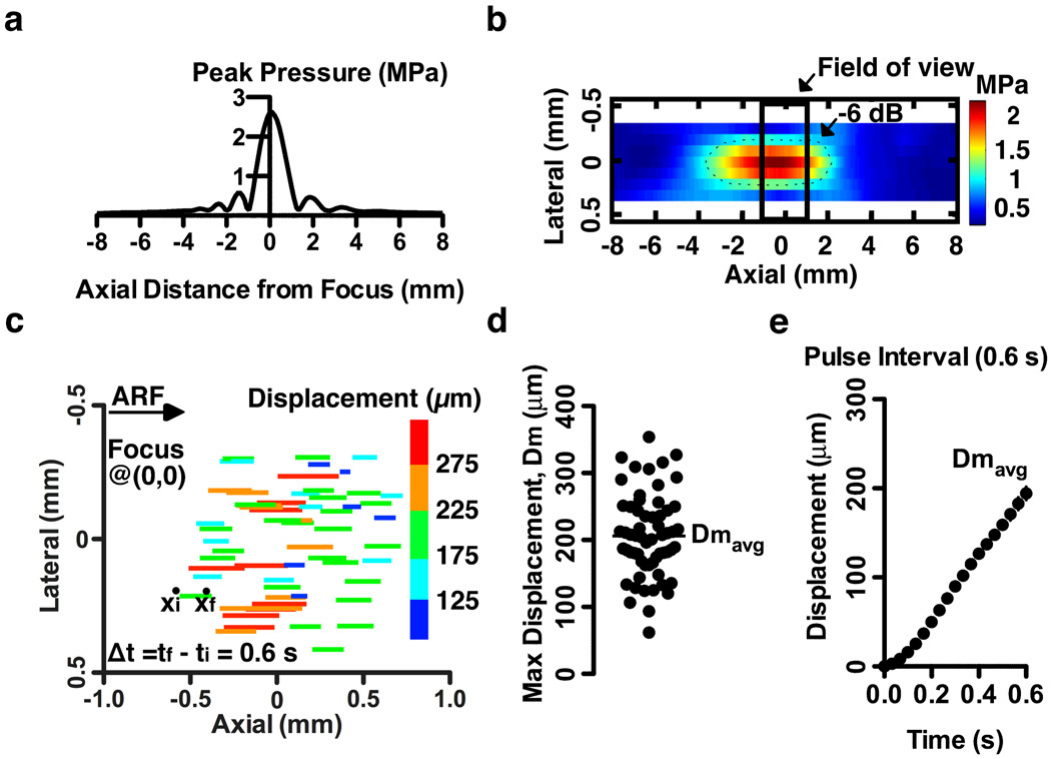
Ultrasound transducer pressure field and bead displacement in a viscous fluid. (**a**) The pressure field of the 10 MHz focused ultrasound transducer at the focal length (19mm) was simulated in Field II software. (**b**) Hydrophone measurements of the acoustic pressure produced by the ultrasound transducer were used to determine the -6 dB lateral beam width (400 μm) and the -6 dB axial beam length (5 mm) at the focus. The microscope field of view is shown. In **b** & **c**, the y-axis is expanded in the interest of clarity. (**c—e**) The microscope field of view was positioned on the beam focus by centering over the largest bead displacements. The ARF induced displacement of 15 μm beads was analyzed in the S6 viscosity standard (8.743 cP). Bead displacement was measured during the 0.6 s ARF pulse interval, Δt = t_f_-t_i_ where maximum displacement D_m_ was determined at t _f_.

**Fig 3:**
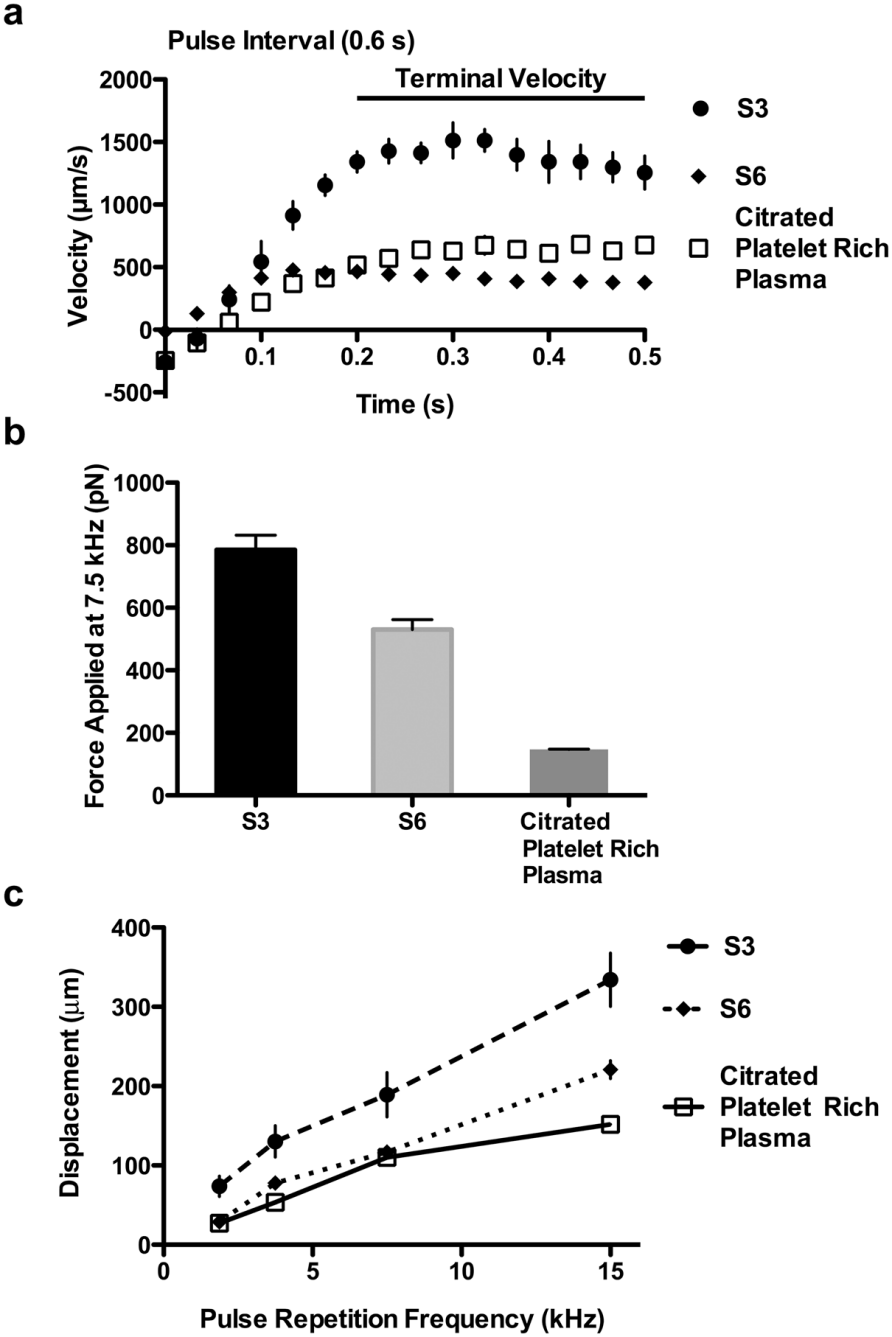
The effects of attenuation and pulse repetition frequency (PRF) on bead velocities and force magnitude. (**a**) Bead terminal velocities under applied ARF at 7.5 kHz PRF in the viscosity standards S6 (8.743 cP) and S3 (4.063 cP), and in citrated platelet rich plasma were measured. Standard error is shown with n = 10 beads. (**b**) Applied ARF magnitude at 7.5 kHz was determined using Stokes’ law and terminal bead velocities. Distinct acoustic attenuations between the fluids account for much of the difference in the applied force in the fluids. At 7.5 kHz the force applied in citrated blood plasma was estimated to be 147 pN. Standard error is shown with n = 10 beads. (**c**) Bead displacement after 0.3 s of pulsing due to applied ARF increased linearly with increasing PRF in the three fluids. Standard error is shown with n = 9 beads.

A focused ultrasound beam was aimed into a 2 mL sample volume container coupled acoustically via ultrasound gel (Fig 1a). In order to determine the focal point and shape of the beam, the pressure field was simulated in FIELD II (Fig 2a) [32]. Pressure was directly measured by hydrophone in a region spanning the analytically determined ultrasound focus (Fig 2b). A 2-D heat map defined the region of maximum pressure and the -6 dB beam boundaries (delineated by the dotted line). The -6 dB lateral beam width was 400 μm, which could be entirely observed within the 10 X microscope field of view (Fig 2b). The -6 dB axial beam length was 5 mm, resulting in a shallow pressure gradient relative to the optical field of view (2 mm in the axial direction), which allowed us to assume that the beads were in a relatively constant pressure field in the direction of wave propagation. Assumption of constant pressure field is supported by velocity data with viscosity standards, where terminal velocities (constant velocities for multiple tenths of a second during the pulse) are reached (Fig 3a).

### Region of bead displacement

The bead displacements during application of a 7.5 kHz ARF impulse were tracked by video microscopy (Fig 1b). Bead displacements were measured by recording trajectories in a S6 viscosity standard (8.743 cP at room temperature). Maximum bead displacement (D_m_) induced by an ARF impulse relative to position in the pressure field is shown in Fig 2c. Since the ultrasound beam has an approximately Gaussian power distribution with respect to its axis, we anticipated that there would be a gradient in the bead displacements across the microscope's 10 X objective field of view. As expected bead displacements were largest at the center of the transducer focus and smallest near the edges (Fig 2c). The average of the maximum bead displacement due to application of an ARF impulse (Dm_avg_) was 206.2 +/- 62.6 μm (n = 60). The distribution of D_m_ across the field of view (1 x 2 mm) was approximately Gaussian (Fig 2d). Average bead displacement during the pulse approached a terminal velocity within 0.1 s of pulse initiation (Fig 2e).

### Bead sedimentation

To control for bead sedimentation effects in clotting plasma prior to reaching the gel-point, we measured bead sedimentation velocity in citrated plasma. Bead sedimentation velocity was determined experimentally in anti-coagulated platelet rich plasma (PRP) by measuring settling time. The 15 μm polystyrene beads settled at an average velocity of 3.84 +/- 0.15 μm/s (n = 250). The distance a bead fell during a 0.5 s pulse train was approximately 1.92 μm. The bead sedimentation distance constituted a 0.5% change in bead position within the -6 dB lateral beam depth (400 μm). Because bead sedimentation was small relative to the beam diameter, and sedimentation would be abolished once the clot was past the gel-point, it was assumed to negligibly influence observed bead trajectories.

### Magnitude of force application

The magnitude of ARF applied to beads in clotting blood plasma was estimated by measuring displacements in citrated PRP and correlating motion to force using Stokes’ law (Eq 1). Bead motion in citrate anticoagulated PRP was compared to that in viscous standards. Terminal bead velocities during ARF application were measured (Fig 3a) and Stokes’ law for drag force was used to calculate the force applied to the beads (Fig 3b) [38]. Stokes' law is:

F=6 π μ Rv (1)

where *F* is the force applied to the beads, *μ* is the fluid viscosity, *R* is the bead radius, and *v* is the bead terminal velocity [39]. The bead terminal velocities in the viscosity standards S6 (8.743 cP at room temperature) and S3 (4.063 cP at room temperature) and citrated PRP (1.6 cP at room temperature) [35] were 429.4 +/- 57.2 *μ*m/s, 1367.4 +/- 256.4 *μ*m/s, and 646.5 +/- 120.4 *μ*m/s respectively. From Stokes' law (Eq 1) it was determined that the force magnitude applied at 7.5 kHz pulse repetition frequency on the beads in S6, S3, and citrated PRP was 530.7 +/- 70.7 pN, 785.4 +/- 147.3 pN, and 147.0 +/- 27.2 pN respectively (Fig 3b). The differences in the applied force on the beads may reflect differences in the degree of ultrasound attenuation between the three fluids, so attenuation of the viscosity standards was measured.

### The relationship between attenuation and force magnitude

All materials attenuate acoustic waves [22]. The attenuation in S6 and S3 viscosity standards and in the agarose elastic standard was measured to determine the influence of attenuation on force experienced by the beads. Acoustic signal amplitudes in the viscous and elastic standards were measured to determine attenuation and force applied to the beads for each material.

The attenuation coefficient of a material is described by the equation

$$\frac{V_{f}}{V_{i}}=10^{-\alpha lf/10}$$ (2)

where V_i_ is the voltage of the signal before entering the material, V_f_ is the voltage of the signal after traveling a distance (*l*) through the material, *f* is the frequency of the signal, and α is the attenuation coefficient. The transducer frequency (*f*) was 10 MHz and the acoustic path length (*l*) in the sample was 1.9 cm.

Change in signal voltage due to signal path length and material attenuation was determined by the average absolute value of the waveform. In all samples signal voltage decreased linearly with thickness. With the voltage attenuation data, a version of Eq 2 specific for each thickness of material was established. The system of equations was solved for the attenuation coefficient of the material using the Matlab function lsqnonlin. The attenuation of water was set to a previously reported value (0.0022 *dB·cm* ^*-1*^·*MHz* ^*-1*^) [37]. The attenuation coefficient of 0.5% agarose was found to be 0.0109 *dB·cm* ^*-1*^·*MHz* ^*-1*^. The attenuation coefficients of the S3 and S6 viscosity standards were determined to be 0.0764 *dB·cm* ^*-1*^·*MHz* ^*-1*^ and 0.1056 *dB·cm* ^-1^·*MHz* ^-1^, respectively.

Eq 2, which describes attenuation of ultrasound signal voltage, can be extended to reflect a proportional change in applied force with the change in voltage across a signal path length within the material, such that

$$\frac{F_{f}}{F_{i}}=\frac{V_{f}}{V_{i}}=10^{-\alpha lf/10}$$ (3)

where α is the material attenuation, *F*_*i*_ is the ideal (non-attenuated) force delivered by the transducer, *F*_*f*_ is the force calculated on beads in the material from Stokes’ law (Eq 1) [40], V_i_ is the voltage of the signal before entering the material, V_f_ is the voltage of the signal after traveling a distance (*l*) through the material, and *f* is the transducer frequency. The lower ultrasound attenuation of S3 compared to S6 was consistent with the greater force experienced by beads in the S3 fluid than in the S6 fluid.

To determine the expected force differential between two materials a ratio of Eq 3 can be made. When the settings of applied ultrasound are the same for each material such that the *F*_*i*_ in each material are equal

$$\frac{\frac{F_{material\;1}}{F_{i}}}{\frac{F_{material\;2}}{F_{i}}}=\frac{F_{material\;1}}{F_{material\;2}}=10^{-\alpha_{1}lf/10}/10^{-\alpha_{2}lf/10}$$ (4)

where *F*_*material 1*_ is the force on beads in a material, *F*_*material 2*_ is the force on beads in a second material, α_1_ and α_2_ are the attenuation coefficients for the respective materials, *f is* the transducer frequency and *l* is the acoustic path length.

The expected force differential between S6 and S3 viscosity standards was determined from the attenuation coefficients measured previously using Eq 4 such that

$$\frac{F_{S6}}{F_{S3}}=\frac{10^{-\frac{\alpha_{S6}lf}{10}}}{10^{-\frac{\alpha_{S3}lf}{10}}}=\frac{0.630}{0.715}=0.881$$ (5)

The statistically bounded range of the F_S6_ to F_S3_ ratio using bead displacement analysis by Stokes’ law was

$$\frac{F_{S6}}{F_{S3}}=\frac{530.7\pm 70.7\;pN}{785.4\pm 147.3\;pN}=\;0.493\;–\;0.942$$ (6)

Since the ratio predicted by the fluid attenuations was within the Stokes analysis range, the agreement of the force and attenuation measurements suggested that fluid attenuation accounted for the difference in force (F_f_) experienced by the beads in their respective fluids.

The lower force experienced by beads in citrated PRP relative to S3 or S6 standards predicts that blood plasma has a greater attenuation than S3 and S6 viscous standards, or even a 0.5% agarose gel. Previously reported attenuation coefficients (α) for unclotted and clotted blood plasma are 0.104 *dB·cm* ^*-1*^·*MHz* ^-1^ and 0.126 *dB·cm* ^*-1*^·*MHz* ^-1^ respectively [41]. The expected force differential between clotted and unclotted plasma was determined from the attenuation coefficients measured previously using Eq 4 such that

$$\frac{F_{clotted}}{F_{unclotted}}=\frac{10^{-\frac{\alpha_{clotted}lf}{10}}}{10^{-\frac{\alpha_{unclotted}lf}{10}}}=\frac{0.575}{0.634}=0.907$$ (7)

As the relative difference between the estimated force acting on a bead in clotted and unclotted plasma differs by less than 10% based on the relationship between force and attenuation described in Eq 4, we chose to use the average of the two coefficients for all plasma conditions. Therefore, given the assumption of constant attenuation coefficient 0.115 *dB·cm* ^*-1*^·*MHz* ^-1^, a constant force of 147 pN at 7.5 kHz PRF was assumed for all blood plasma material property measurements with the optical ARF technique.

### The relationship between pulse repetition frequency (PRF) and force magnitude

Based on previous ARF theory [29], we hypothesized that varying the PRF (the number of ultrasound impulses applied within one second) would adjust the force applied to the beads and lead to a corresponding modulation of bead displacements. To test our hypothesis the PRF was varied in the S3 and S6 viscosity standards and in citrated PRP (Fig 3c). Material stiffness was correlated with PRF and bead displacement by the equation

$$D_{m}\propto \;\frac{\text{PRF}}{S}$$ (8)

where *D*_*m*_ was the maximum displacement of the bead after the ARF impulse, *S* was material stiffness, and *PRF* was the pulse repetition frequency of the material [28]. Reducing the *PRF* resulted in a linear reduction in the bead displacements, consistent with theory (Eq 8).

### Viscoelastic measurement of platelet poor plasma (PPP) clotting

**Fig 4:**
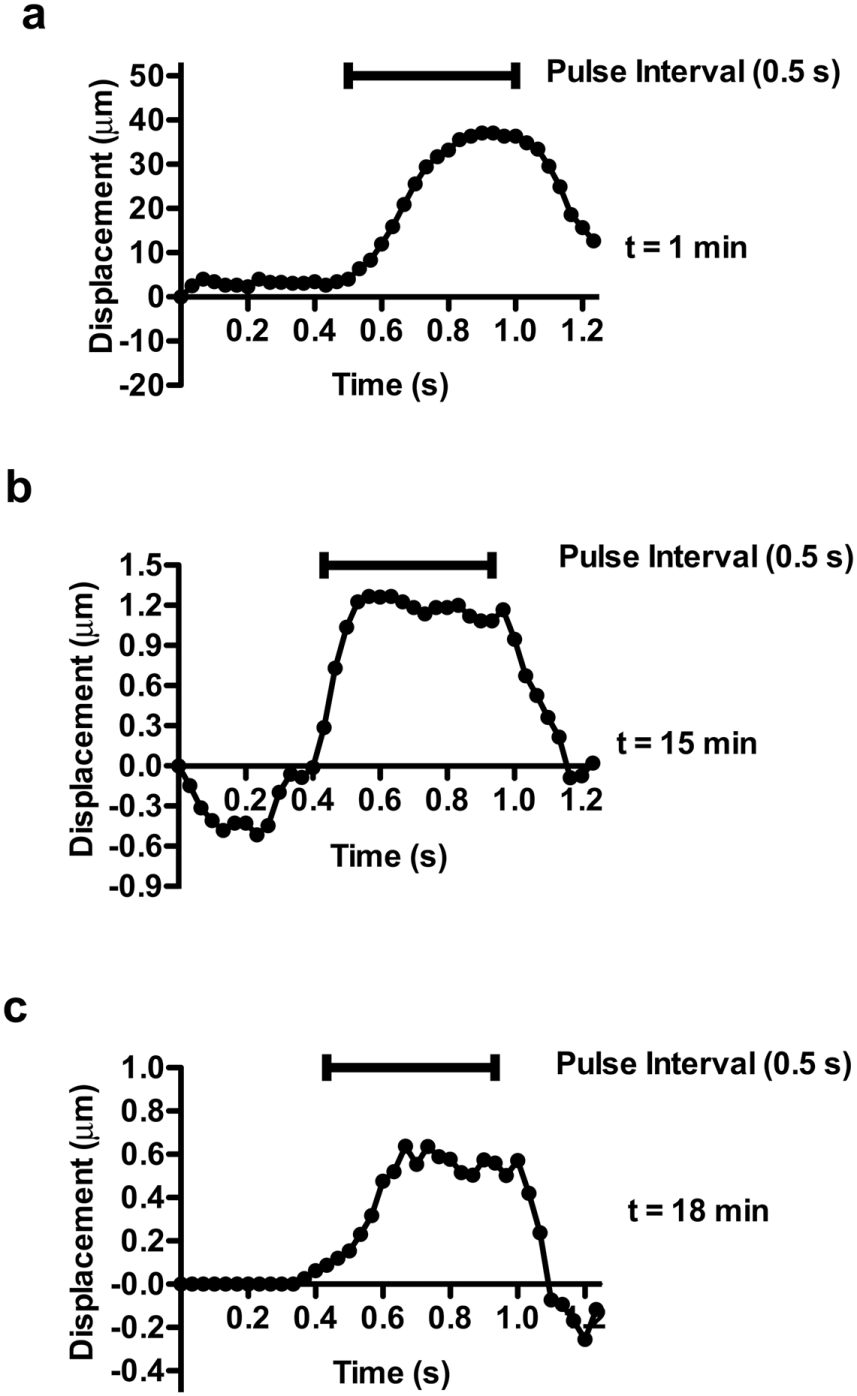
Characteristic bead displacement due to applied ARF at 7.5 kHz in clotting platelet poor plasma. (**a**) At t = 1 min the bead displaced 40 μm during a 0.5 s pulse interval and recoiled after the pulse stopped. (**b**) At t = 15 min the bead displaced 0.9 μm during the pulse interval and recoiled after the pulse stopped. (**c**) At t = 18 min the bead displaced 0.5 μm during the pulse interval and recoiled after the pulse stopped. Optical noise was reduced by smoothing with a 3 data point moving average filter.

Fibrin contributions to stiffness were assessed by removing platelets from plasma through centrifugation to generate PPP. Bead displacement due to ARF pulsing decreased over the course of 17 min (Fig 4a–4c). Signs of increasing elasticity developed early after clot initiation; within 1 min of kaolin activation beads recoiled after removal of an ARF impulse (Fig 4a). Based on bead velocities, the PPP sample at 1 min had a *Re* ~ 0.07 [39], suggesting that the sample was experiencing significant viscous damping of the developing elastic response, characteristics of a viscoelastic fluid. At later time points, the Reynolds number reduced further (*Re* < 0.002) as the viscosity increased and the velocity of the beads was reduced (Fig 4b and 4c). At 15 (Fig 4b) and 18 (Fig 4c) min the beads reached a plateau of maximum displacement during the ARF impulse, recoiling 100% after force application stopped, indicative of a high degree of material elasticity.

### Viscoelastic model fitting of platelet poor plasma

**Fig 5:**
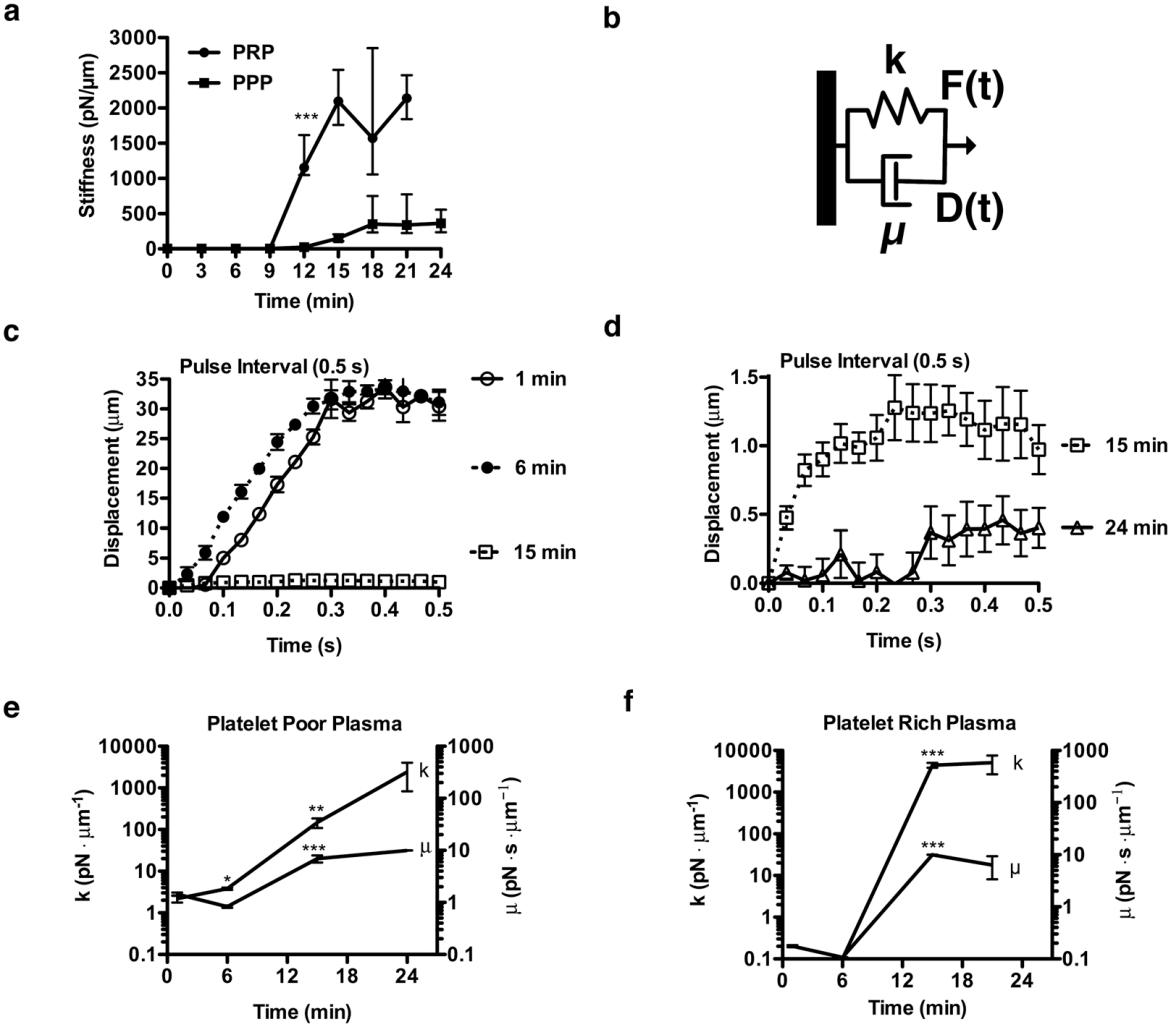
Characteristic mechanical properties of clotting platelet rich (PRP) and platelet poor plasma (PPP) as assessed by application of ARF at 7.5 kHz. (**a**) Clot stiffness (S), S(t) = F_plasma_/Dm_avg_, of clotting PRP and PPP was determined. Bead displacements (n = 5) were averaged to compute stiffness, which is displayed with 60% quartiles (***, p<0.0005 between PPP and PRP from t > 12 min). (**b**) In the Kelvin-Voigt model, which predicts the displacement D(t) response of viscoelastic materials to applied force F(t), a viscous element (dashpot) is in parallel with an elastic element (spring). The parameters k and μ describe the elastic and viscous properties respectively. (**c-e**) The Kelvin-Voigt model was fit to bead displacement data in clotting PPP (**c**) where displacement decreased abruptly between 6–15 min. (**d**) From 15–24 min the model was fit to bead displacement data and displacement continued to decrease. At all time points, in contrast to measurements in the viscous fluids, the displacement approached a maximum. Standard error is shown with n = 5 beads per time point. (**e-f)** Kelvin-Voigt viscous (μ) and elastic (k) parameters of PPP and PRP were determined. Standard error is shown with n = 5 beads per time point (*, p<0.05)(**, p<0.006)(***,p<0.0005). (**e**) During clotting in the PPP sample the Kelvin-Voigt viscous (μ) and elastic (k) parameters increased 1107 fold and 6.9 fold respectively. (**f**) During clotting in the PRP sample the average fold difference of viscous (μ) and elastic (k) parameters of the clot as compared to PPP between 15 and 21 min were 1.3 fold and 11 fold larger, respectively.

Clot stiffness in PPP was determined by the equation:

$$S=\;\frac{F_{plasma}}{D_{m}}$$ (9)

where *S* is the clot stiffness, *F*_*plasma*_ is the ARF applied to beads in plasma, and *D*_*m*_ is the average of bead maximum displacements after application of 0.5 s of an ARF pulse (Fig 5a). As a form of Hooke’s law, the inverse relationship between applied force and resultant displacement defines stiffness (*S*) (Eq 9) [42]. Acoustic radiation force has been used in defining material stiffness in previous work [29]. The clot appeared to have near constant stiffness from 0–9 min and then stiffness increased 78-fold between 9 and 18 min. In order to quantify the changes in viscous and elastic components of the plasma during clotting the displacement data was fitted with the Kelvin-Voigt model.

The Kelvin-Voigt model consists of a spring and a dashpot in parallel (Fig 5b). The spring constant, viscous dashpot constant, and the equation that defines the system together describe the displacement of the point of interest due to a given force. To evaluate the viscoelastic properties the characteristic equation for the Kelvin-Voigt model was fit to the displacement data during the application of a pulse train (see the Methods bead tracking section). The Kelvin-Voigt model captures the coupled effects of viscosity and material elasticity on force-induced material displacements and has been widely used in biological materials modeling [43].

Assumptions of the Kelvin-Voigt model include application of constant force, which is appropriate if the analysis is limited to the displacements measured during the pulse train interval. The force applied on the blood plasma sample can be considered constant during a pulse train interval because the ratio of the time constant of blood plasma viscoelastic response (~0.01 s) [44–46] to the pulse repetition period (~10^-4^ s/cycle) is so large. Tissue (or blood clot) movement is negligible between each impulse, and therefore during the microsecond pulse train application the force is effectively constant on the tissue. Momentum of the bead, because of low Reynolds number, is assumed negligible and therefore not accounted for in the model.

The Kelvin-Voigt model was fit to bead displacement during force application to assess the viscoelastic behavior of blood [28,47,48]. Within a minute of PPP clot initiation spring-like behavior (displacement plateau during impulse) was evident and increased at all times measured (Fig 5c and 5d). In contrast, viscous fluid-like behavior (constant velocity during impulse) was observed with bead motion in the S6 viscosity standard at all time points measured. The elastic parameter k of S6 fluid, as expected, was near zero (S4 Fig). Viscous (μ) and elastic (k) parameters of PPP determined by the model at 24 min were 9.999 +/- 0.001 *pN·s·μm* ^*-1*^ and 2430 +/- 1600 *pN·μm* ^*-1*^ respectively (Fig 5e). This is an increase in viscous and elastic parameters of 6.9 fold and 1107 fold respectively from unclotted PPP. The clot properties changed most significantly between 6 and 15 min, when elasticity (k) rapidly increased.

### Platelet depletion and clot stiffness

Stiffness of PPP samples was compared to that of PRP to assess platelet contributions to clot stiffness (Fig 5a). PRP increased clot stiffness 6.9 fold relative to PPP. Where significant elasticity developed, beads in both PPP and PRP recoiled after ARF induced displacement (Fig 4), a hallmark of reversible elastic deformation. Viscous (μ) and elastic (k) parameters determined by the model at 21 min in PRP are 6.364 +/- 2.988 *pN·s·μm* ^*-1*^ and 5127 +/- 2440 *pN·μm* ^*-1*^ respectively (Fig 5f). To assess the clot properties of PRP after initial hemostasis, the average fold difference of viscous (μ) and elastic (k) parameters of the clot as compared to PPP between 15 and 21 min were 1.3 fold and 11 fold larger, respectively. In PRP, elasticity and viscosity both developed most significantly between 9 and 15 min, paralleling the kinetics of viscosity and elasticity changes in clotting PPP. Video of PRP clotting is available in S1 Video.

### Measuring stiffness ratio of agarose elastic standard to blood plasma

For interpretation of the measurements made by the optical ARF method relative to standard material property testing techniques, the stiffness of PRP was related to that of a 0.5 wt % agarose elastic standard. Both PRP and agarose have been measured by standard material property testing techniques such as the parallel plate rheometer. Stiffness comparison required the previously determined attenuation coefficients for plasma and agarose. The range of reported shear modulus of 0.5 wt% agarose is 1400–4530 Pa [49–52]. Previously reported shear moduli of PRP range from 406–600 Pa [11,53,54]. The ratio of the shear moduli of 0.5 wt% agarose to PRP as previously reported ranges from 2.3–11.2.

The ratio of stiffness of 0.5 wt% agarose and PRP measured by our optical ARF technique was compared directly to the ratio of previously reported shear moduli in order to validate the material property measurement capabilities of the optical ARF technique.

The expected force differential between PRP and agarose was determined from the attenuation coefficients measured previously using Eq 4 such that

$$\frac{F_{plasma}}{F_{agarose}}=\frac{10^{-\frac{\alpha_{plasma}lf}{10}}}{10^{-\frac{\alpha_{agarose}lf}{10}}}=\frac{0.588}{0.953}=0.617$$ (10)

With the difference in force application between PRP and agarose determined, the displacements of beads in each material were measured. The beads in PRP were displaced 8.25 +/- 0.91 μm and those in agarose were displaced 1.369 +/- 0.13 μm. In order to compare the stiffness of the plasma to that of agarose the following equation was used

$$\frac{S_{agarose}}{S_{plasma}}=\frac{F_{agarose}}{F_{plasma}}\times \frac{d_{plasma}}{d_{agarose}}$$ (11)

where S_agarose_ is the stiffness of agarose, S_plasma_ is the stiffness of plasma, d_agarose_ is the bead displacement in agarose, and d_plasma_ is the bead displacement in plasma. Using Eq 11 the 0.5 wt % agarose gel was approximately 9.8 fold more stiff than a clotted PRP sample. This was within the ratio range (2.3–11.2) of the shear moduli reported previously for 0.5 wt% agarose and clotted PRP in independent determinations.

### Measurement technique sensitivity to strength of clotting stimulus

**Fig 6:**
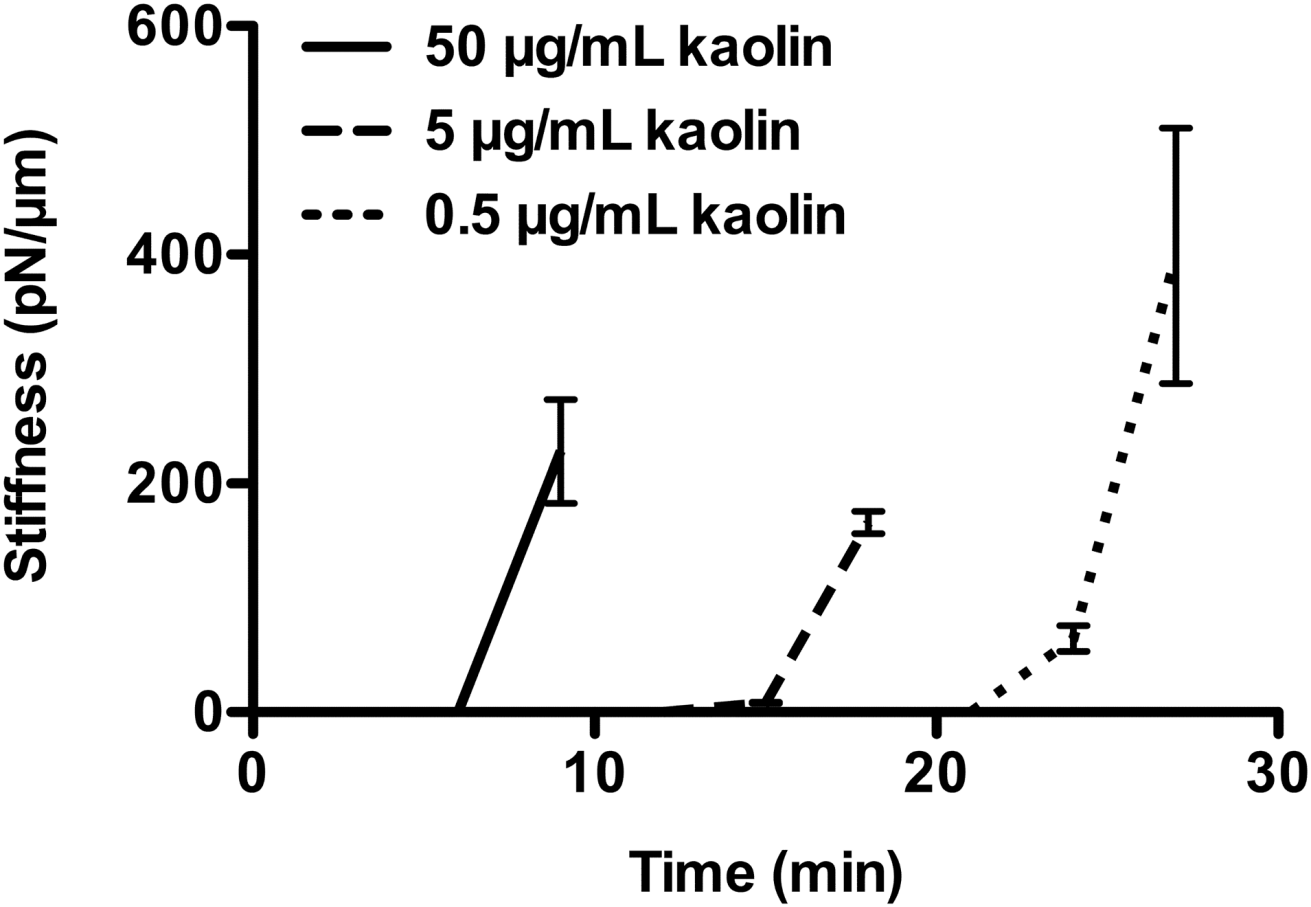
Sensitivity of optical ARF clot stiffness assessment to strength of kaolin clotting stimulus. Clot stiffness of PRP stimulated with 0.5, 5, and 50 μg/mL kaolin was measured by optical ARF. Optical ARF detected kaolin concentration dependent kinetics of clot stiffness. Standard error is shown with at least n = 5 beads per time point.

Clotting of PRP was stimulated over a range of kaolin concentrations to assess the sensitivity of the optical ARF based clot stiffness assessment technique to strength of clotting stimulus (Fig 6). The measurements showed the capability of the ARF based bead-tracking technique to detect differences in kinetics of stiffness development that occur with variation in coagulation stimulus.

## Discussion

In this study we describe an application of acoustic radiation force (ARF) to measure blood plasma clot stiffness. In this technique microbeads were embedded in the plasma to serve as coupled force transducers and strain gauges. ARF induced micron-scale displacements of acoustically reflective beads that were tracked by video microscopy. Bead forces were calibrated in viscosity standards to quantify viscoelasticity changes in blood plasma due to fibrin network formation. The motion of beads under ARF was initially dominated by blood plasma viscosity and later, as clotting progressed, was marked by increased elasticity. Comparison of the stiffness ratio of plasma and agarose gels measured by optical tracking of ARF-induced bead displacement to their previously reported shear moduli provided a point of reference to other rheometric techniques. Optical measurement of ARF-induced bead displacement (optical ARF) to assess blood plasma viscoelasticity was sensitive to biologically relevant characteristics of blood clotting such as strength of activation stimulus and platelet stiffness contributions.

ARF generation requires ultrasound scatterers in the material to reflect and absorb energy. While the material itself experiences force, additional scatterers, such as embedded beads, significantly increase force application. ARF results from the millisecond integration of thousands of individual acoustic pressure waves into what is effectively a sustained impulse. Pressure wave integration occurs because the pulse repetition period (~10^-4^ s/cycle) is much smaller than the time required for tissues to relax between acoustic pulses (~1 ms) [44–46]. Key to the utility of beads as force transducers are their greater attenuation of acoustic energy compared to the suspending medium, a difference of approximately 6-fold for polystyrene beads relative to plasma [55]. The bead radius is another significant determinant of optical ARF, as surface area is directly proportional to the resultant force [56]. A related application of ARF to blood clot stiffness estimation, sonorheometry, relies on the red blood cells at the beam focal point to reflect ultrasound and act as force transducers [28–30].

While material attenuation and bead radius influence the transmittal of ARF, ultrasound wave characteristics also affect the resultant force. Variation of pulse repetition frequency (PRF) was shown to proportionally modulate the force on the bead. By varying the PRF, the force per bead could be varied from 10–294 pN. These forces are on the order of those required for rupture of a fibrin fiber bond (200 pN) or to stretch a monomer in a fibrin fiber to 100% strain (140 pN) [57,58]. Since rupture of fibrin fibers in a network requires greater force than that needed to rupture a single fibrin bond, we assume the forces applied by ARF in this study to be largely non-destructive to the fibrin network, allowing evaluation of its viscoelasticity.

The assessment of plasma clot viscoelasticity by optical ARF was further validated by comparison with an elastic standard, agarose. Stiffness measurements of PRP, PPP and agarose were compared shear moduli determined in conventional rotational rheometers. In our case, agarose was determined by optical ARF to be 9.8-fold stiffer than PRP. This stiffness ratio was used to estimate a shear modulus ratio of agarose to PRP. Assuming the relationship was approximately proportional, the known shear modulus of 0.5% agarose ranging from 1400–4530 Pa would predict a shear modulus range of 143–462 Pa for PRP [49–52,59]. The estimate of the shear modulus for PRP was on the order of the 406–600 Pa range estimated in previous reports at equivalent times [11,53,54]. The 6.9 fold stiffness ratio of PRP to PPP measured by optical ARF was also of similar proportion to the reported stiffness ratio of 8.6 measured with the parallel plate rheometer [11]. Optical ARF estimates of stiffness therefore scales closely to a classic rheologic test and therefore can be extrapolated, within appropriate limitations, to a wider range of biologic testing of plasma blood clotting mechanics.

With the use of optical ARF there are several considerations that may confound the interpretation of the results. For instance, Brownian motion of the embedded bead could conceivably influence the estimation of the ARF-induced viscoelastic response. However, the diffusion coefficient of a 15 μm diameter bead in blood plasma was estimated to be 2.6 × 10–^14^ *m* ^2^/*s*, and therefore the average bead would move only 0.33 μm/s [39,60,61]. Brownian motion was therefore considered to contribute negligibly in unclotted blood plasma relative to ARF induced bead displacement (>100 μm). Once a fibrin network has formed the bead diffusivity would drop even further. A related factor that could influence the interpretation of bead motion is the relative size of the beads compared to the fibrin network pore size and potential molecular interactions between bead and fibrin. However, polystyrene beads have been shown to non-specifically adhere to fibrin fibers [21] and the bead size (15 μm) is more than an order of magnitude larger than estimates of the fibrin mesh pore size (0.6 μm) [62], suggesting that the beads are likely trapped in the gel by adhesion and physical entanglement. Another potential concern is that bead motion under the influence of shear waves would be too fast to be captured at standard video rates. However, the maximum velocity at which beads moved when unconstrained in plasma was 600 μm/s, thus their frame to frame displacement was ~ 20 μm—a distance easily resolvable by microscopy. A final point of consideration is the potential effect of the beads on the coagulation process. Embedding beads in a blood sample reduced time to clot and increased clot stiffness in a sonorheometry measurement (see S5 Fig), However, in our study, the presence of the beads was a constant and thus all samples experienced the same level of contact-pathway stimulation.

The detection of viscoelastic material properties by ARF-induced bead motion has several experimental limitations. For one, a microscope-based bead tracking approach has an inherent stiffness detection limit defined by video resolution and the optical signal to noise ratio; i.e, once the gel becomes very stiff relative to the force on the bead, motion stops. Further increases in gel stiffness are undetectable. However, improved signal detection can be achieved by increasing transducer power to increase bead displacement or by raising microscope magnification to increase spatial resolution. Increases in plasma clot turbidity, which becomes a significant optical limitation after 45 minutes, can also reduce bead displacement measurement accuracy. In this study, we used fluorescent beads to allow optical ARF analysis at advanced stages of PRP clot development (~1 h).

The mechanics of blood clots is complex. One of the more intriguing features of fibrin networks is their strain-hardening characteristic, or increased material stiffness with application of large strains (>10–100%) [58,63–65]. A component of the strain hardening is thought to be a consequence of network deformation such that the slack is essentially taken out of the fibrin fibers. In platelet-rich plasma clots strain hardening does not occur [63], possibly by platelets pre-tensioning the network. Consequently, at low strains a PRP clot will have a larger elastic modulus than a PPP clot. But at progressively higher strains the difference between the two decreases as the PPP clot strain hardens, eventually becoming almost equivalent to PRP at strains of 20% and greater. Rotational rheometers such as thromboelastography (TEG) and rotational thromboelastometry (ROTEM), which introduce strain at levels of 8–16%, could therefore potentially strain-harden of blood clots as they assess viscoelasticity. Under conditions where strain-hardening occurs, platelet contributions to clot stiffness may be less evident [53,66,67].

When PRP and PPP clots have been compared in the TEG assay, it has been observed that the stiffness ratio varies from 1.2–4 fold using TEG maximum amplitude (MA) assessment, depending on laboratory [68–71]. In contrast, the optical ARF method described here indicated the presence of platelets increased the clot stiffness by 6.9 fold, which was consistent with a previous study using ARF in whole blood [30]. The relative softness of the ARF probe described here, where induced strains were 2% or less, may therefore be useful in the analysis of platelet contributions to fibrin network stiffness, as sub-optimal platelet function would conceivably be more evident in the absence of fibrin network strain hardening.

*Ex vivo* measurements of clotting, such as the optical ARF method described in this study, almost all suffer a potential limitation in accuracy with regard to inferences of coagulopathies *in vivo* due to the fact that the blood sample is clotted under quiescent conditions. Since blood clot formation under stasis may increase fibrin and decrease platelet concentration as compared to *in vivo* arterial clots [72,73], it is important to qualify interpretations of clot mechanics depending on whether the test is based on quiescent or shear conditions, and additionally how the specific magnitude of shear might influence clotting; i.e., is the shear arterial or venous? With that consideration in mind, static condition measurement techniques, such as optical ARF, may be more appropriate when assessing coagulopathies characterized by venous flows, trauma, or surgical bleeding.

Optical ARF viscoelasticity measurements as described in this study appeared to be sensitive to both the contribution of platelets to clot stiffness and the level of clotting stimulus. Consequently, the approach could potentially be employed for studies on early stages of clot formation, which likely involves activation of the platelet contractile apparatus in addition to contributions of fibrin polymerization [74]. In addition, ARF measured platelet contributions to stiffness in a 3D clot matrix rather than on a surface, differentiating it from direct measures of platelet contractility such as micropost and AFM assays that typically require testing of isolated platelets on a substrate [75,76]. During clot formation, platelet contractile activity is translated to larger length scales through fibrin filaments and concurrent platelet aggregation. The ARF method we've described here may be useful in characterizing how single platelet force generation ultimately leads to the generation of tensioned fibrin networks and clot stiffness.

## Supporting Information

S1 Fig The sample holder. The field of view for the 10 X objective is 2000 μm x 1200 μm. The shape of the PDMS mold works to reduce volume requirements for the blood sample, while the PDMS material properties allow it to act as an ultrasonic absorber reducing container reflections.
(TIFF)

S2 Fig Bead displacement in optical focal planes. To find the focal plane at the center of the ultrasound beam the bead displacement during ultrasound pulsing was measured at multiple focal planes within the sample. The plane at which measurements were made in the sample was chosen at 1.3 mm above the bottom of the sample holder. At 1.3 mm backflow, bead displacement opposite to the direction of ultrasound beam application, likely due to convection, was not present.
(TIFF)

S3 Fig Schematic of setup for attenuation measurement in fluids. In a water bath, a signal transmitting transducer was fired into the fluid sample at 3 path lengths of fluid. The signal receiver recorded the resulting waveform. For agarose gel samples, slabs of gel were placed between the transmitter and receiver in the place of the flask, with 3 thicknesses. The change in amplitude of the waveform between the path lengths of fluid or gel were used to calculate the attenuation of each material.
(TIFF)

S4 Fig Viscoelastic measurement of viscous fluid S6. (**a**) The stiffness (S) of the material was determined with the force applied on beads in S6 (F_S6_) and the bead displacement during a 0.6 s pulse interval (Dm_avg_) by the equation S = F_S6_/Dm_avg_. (**b-c**) The Kelvin-Voigt model was fit to the displacement during the pulse interval from which (**b**) viscous parameter μ and (**c**) elastic parameter k were calculated, which showed, as expected, a viscous-dominated mechanical response.
(TIFF)

S5 Fig Influence of polystyrene beads on clot stiffness. The addition of beads to whole blood samples as measured by sonorheometry resulted in increased kinetics of clot stiffness and maximum clot stiffness as compared to whole blood samples without beads.
(TIFF)

S1 Video Plasma clotting in optical acoustic radiation force clot stiffness assessment. Polystyrene microbeads (15 μm) in platelet rich plasma were displaced due to application of the acoustic radiation force. Bead displacement was reduced with increasing plasma clot viscoelasticity over the course of minutes. Clotting of platelet rich plasma was initiated with 5 μg/mL kaolin.
(MOV)
