# Supplementary figures and images for: Integration of Acoustic Radiation Force and Optical Imaging for Blood Plasma Clot Stiffness Measurement
Source: PLoS One. 2015 Jun 4;10(6):e0128799. doi: 10.1371/journal.pone.0128799 (PMC4456080; doi:10.1371/journal.pone.0128799)

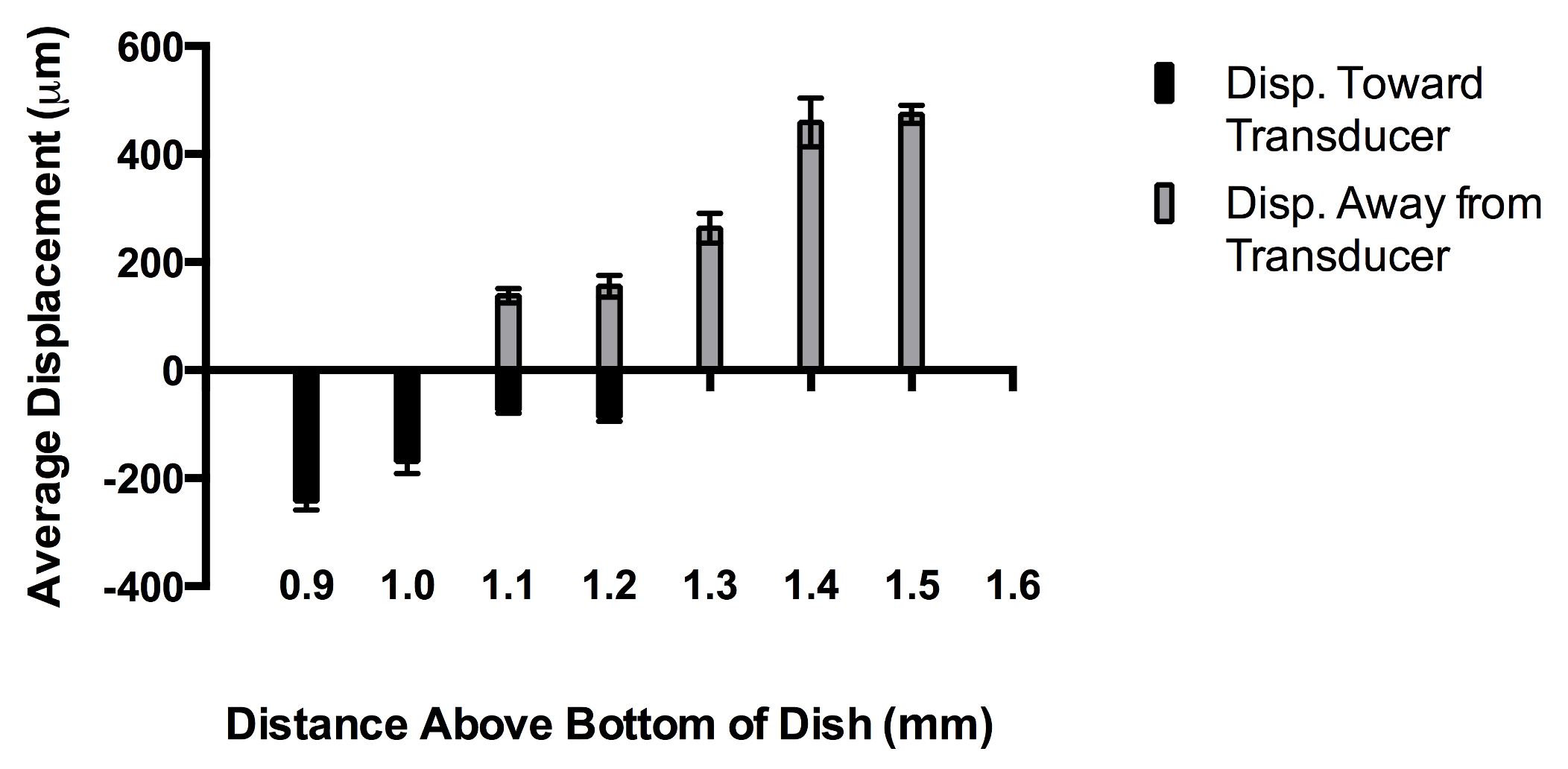

Supplement: S2 Fig — To find the focal plane at the center of the ultrasound beam the bead displacement during ultrasound pulsing was measured at multiple focal planes within the sample. The plane at which measurements were made in the sample was chosen at 1.3 mm above the bottom of the sample holder. At 1.3 mm backflow, bead displacement opposite to the direction of ultrasound beam application, likely due to convection, was not present. (TIFF) [file pone.0128799.s002.tiff]

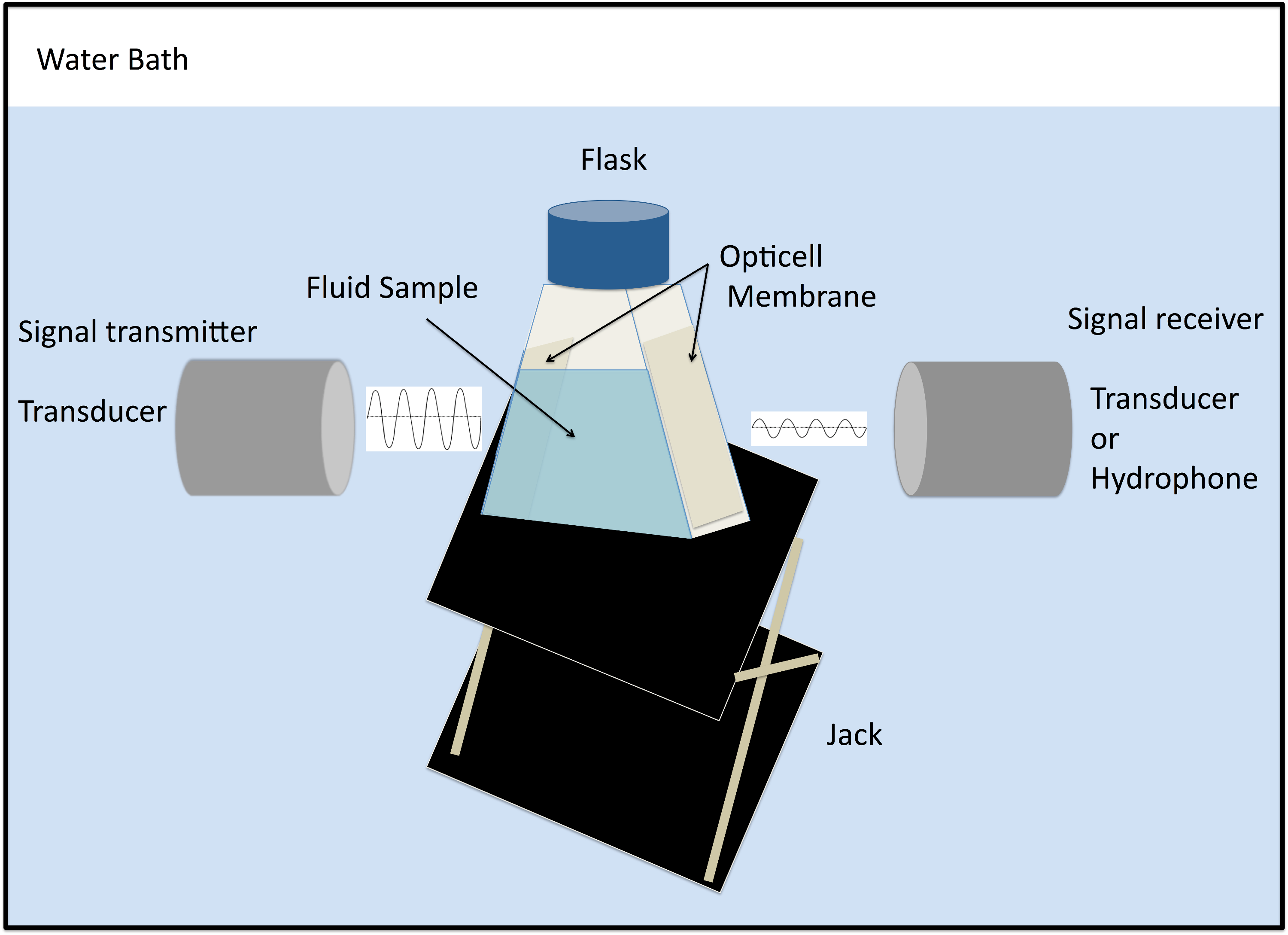

Supplement: S3 Fig — In a water bath, a signal transmitting transducer was fired into the fluid sample at 3 path lengths of fluid. The signal receiver recorded the resulting waveform. For agarose gel samples, slabs of gel were placed between the transmitter and receiver in the place of the flask, with 3 thicknesses. The change in amplitude of the waveform between the path lengths of fluid or gel were used to calculate the attenuation of each material. (TIFF) [file pone.0128799.s003.tiff]

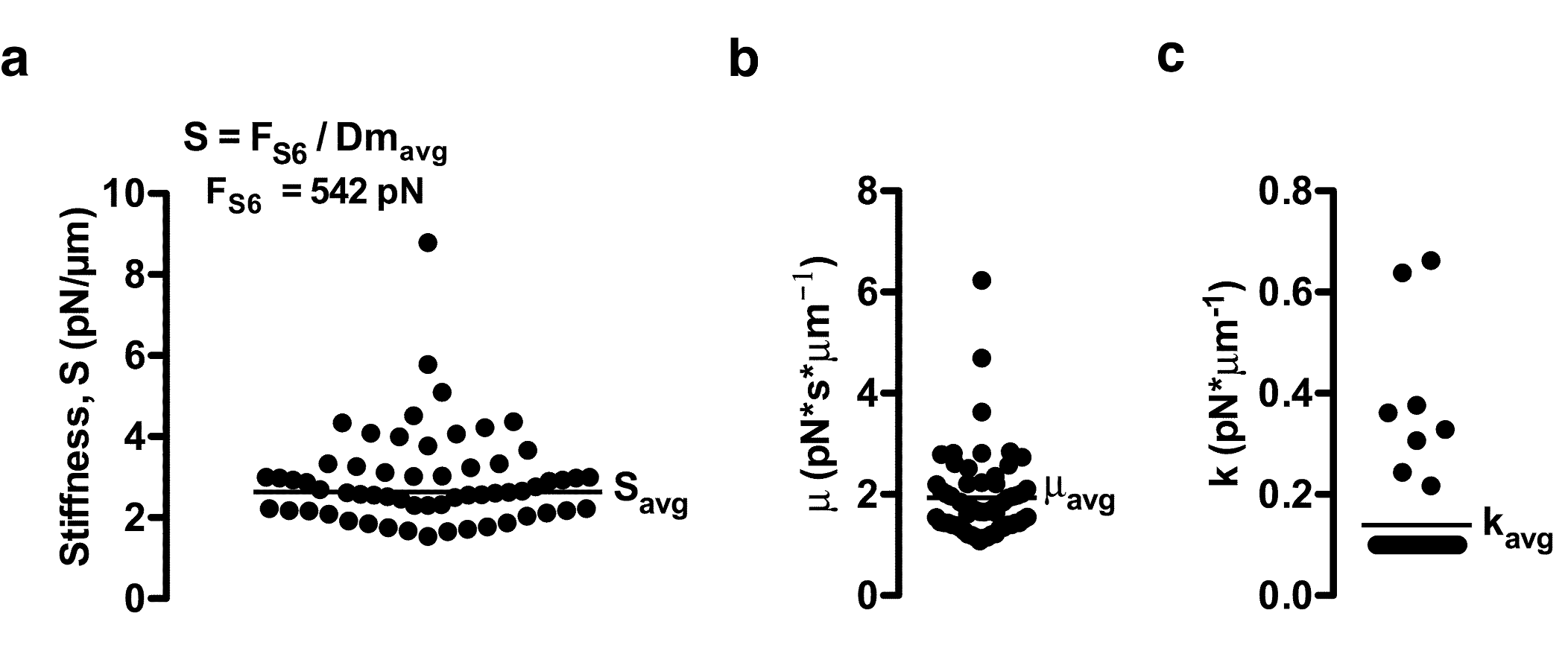

Supplement: S4 Fig — (a) The stiffness (S) of the material was determined with the force applied on beads in S6 (FS6) and the bead displacement during a 0.6 s pulse interval (Dmavg) by the equation S = FS6/Dmavg. (b-c) The Kelvin-Voigt model was fit to the displacement during the pulse interval from which (b) viscous parameter μ and (c) elastic parameter k were calculated, which showed, as expected, a viscous-dominated mechanical response. (TIFF) [file pone.0128799.s004.tiff]
